# Visual analytics identifies key miRNAs for differentiating peripancreatic paraganglioma and pancreatic neuroendocrine tumors

**DOI:** 10.3389/fendo.2023.1162725

**Published:** 2023-06-13

**Authors:** Jose María Enguita, Ignacio Díaz, Diego García, Tamara Cubiella, María-Dolores Chiara, Nuria Valdés

**Affiliations:** ^1^ Department of Electrical Engineering, University of Oviedo, Gijón, Spain; ^2^ Department of Cancer, Health Research Institute of the Principality of Asturias, Oviedo, Spain; ^3^ Respiratory Tract Tumors, CIBERONC (Network of Biomedical Research in Cancer), Madrid, Spain; ^4^ Institute of Oncology of the Principality of Asturias, University of Oviedo, Oviedo, Spain; ^5^ Department of Internal Medicine, Section of Endocrinology and Nutrition, Cabueñes University Hospital, Gijón, Spain

**Keywords:** paraganglioma, pancreatic neuroendocrine tumors, microRNA, diagnosis, visual analytic

## Abstract

**Introduction:**

Paragangliomas (PGL), a type of neuroendocrine tumor, pose a significant diagnostic challenge due to their potential for unpredictable locations and asymptomatic presentation. Misdiagnosis of peripancreatic PGLs, particularly as pancreatic neuroendocrine tumors (PANNETs), is a pressing issue as it can negatively impact both pre- and post-treatment decision-making. The aim of our study was to identify microRNA markers for the reliable differential diagnosis of peripancreatic PGLs and PANNETs, addressing a crucial unmet need in the field and advancing the standard of care for these patients.

**Methods:**

Morphing projections tool was used to analyze miRNA data from PGL and PANNET tumors present in the TCGA database. The findings were validated using two additional databases: GSE29742 and GSE73367.

**Results:**

Our research uncovered substantial differences in the miRNA expression profiles of PGL and PANNET, leading to the identification of 6 key miRNAs (miR-10b-3p, miR-10b-5p, and the miRNA families miR-200c/141 and miR-194/192) that can effectively differentiate between the two types of tumors.

**Discussion:**

These miRNA levels hold potential as biomarkers for improved diagnosis, offering a solution to the diagnostic challenge posed by these tumors and potentially improving the standard of care for patients.

## 1 Introduction

Paragangliomas (PGLs) are neuroendocrine tumors with high genetic predisposition. They originate from catecholamine-secreting paraganglionic cells that have a widespread distribution in humans. PGL can have or not a hypersecretory phenotype. They are categorized based in their origin as either parasympathetic or sympathetic. Parasympathetic PGLs are typically found near the aortic arch, neck, and skull base, while sympathetic PGLs arise along the paravertebral axis, retroperitoneum, in the abdomen and pelvis, with the largest forming the adrenal medulla. The term “pheochromocytoma” specifically refers to intra-adrenal sympathetic PGLs according to the latest WHO guidelines (1). PGLs are highly heritable, with approximately 50% of cases associated with a germline mutation in one of at least 15 genes, making them the most heritable tumours in humans. They can occur as part of well-established hereditary syndromes, including multiple endocrine neoplasia type 2A and 2B, von Hippel–Lindau syndrome, neurofibromatosis type 1 and familial paraganglioma syndromes, caused by pathogenic variants in genes encoding Ret Proto-Oncogene (RET), Von Hippel Lindau protein (VHL), Neurofibromin 1 (NF1), or components of the succinate dehydrogenase (SDH) complex, respectively.

Given the widespread distribution of paraganglia, PGL can occur at virtually all locations in the body except the brain and bones. The emergence of PGL in atypical locations can cause confusion and result in missed diagnoses (2). The histopathologic diagnosis is straightforward for PGLs that present in an expected location with classic catecholamine excess symptoms. However, missed diagnoses can occur when the PGLs are in unusual locations, the patient is asymptomatic, or the PGLs do not secrete catecholamines.

Diagnosing peripancreatic PGLs that resemble primary pancreatic lesions can be difficult as they often lack typical PGL symptoms (3, 4). This can be further complicated by the fact that around 10% of pancreatic neuroendocrine tumors are associated with hereditary syndromes like multiple endocrine neoplasia type I (MEN1), von Hippel–Lindau syndrome, neurofibromatosis type 1, and tuberous sclerosis complex (TSC). Preoperative diagnosis can be done through fine-needle aspiration and biopsy, but PGLs and PANNET can have similar morphologic characteristics, leading to misdiagnosis (5–8). Accurate diagnosis is crucial for proper pre- and post-treatment decision-making (9). Therefore, differentiating peripancreatic PGLs from PANNETs is a crucial aspect of clinical practice. Our previous study revealed similarities in genetic profiles but differences in microRNA profiles between the two neoplasms (10). The goal of the present study was to identify microRNA markers that enable differential diagnosis of peripancreatic PGL and PANNET.

## 2 Materials and methods

### 2.1 TCGA database

*The Cancer Genome Atlas* (TCGA) provides gene expression measurements and other transcription data, including more than 20,000 mRNA and hundreds of miRNA expression levels for more than 10,000 tumors from 33 different cancer types. In this study we consider gene expression RNAseq data of cancer cohorts identified as primary paraganglioma (abbreviated here as PGL: pheochromocytoma and paraganglioma, n=149) and pancreatic neuroendocrine tumors (PANNET; 7 samples) (11) from the TCGA Hub. Data were downloaded from the *Xenabrowser* portal https://xenabrowser.net/datapages/. For every cohort we merged: (a) data containing experimental measurements using the Illumina HiSeq 2000 RNA Sequencing platform and mean-normalized per gene across all cohorts; and (b) data with miRNA mature strand expression RNAseq. The resulting table including gene and miRNA expression levels in units of log2(RPM+1) (RPM=Reads per million), was curated by dropping genes and miRNA with invalid values, and later merged with clinical metadata (downloadable from https://portal.gdc.cancer.gov). This allowed for the analysis of 294 miRNAs.

### 2.2 Interactive data visualization: morphing projections

We used the *t-Distributed Stochastic Neighbor Embedding* (*t*-SNE) algorithm, which is a highly effective method for dimensionality reduction, to visually depict the analyzed tissues according to their genetic signature (12). This approach organizes the samples spatially based on their similarities in gene and miRNA expression, resulting in a visual map containing clusters of samples that exhibit similar genetic characteristics, thereby providing a comprehensive understanding of the predominant genetic profiles present within the analyzed samples.

For the exploratory analysis of the TCGA data we used an in-house developed application that implements the *Morphing Projections* technique (10). This tool facilitates a user-controlled arrangement of the data to enhance the exploration process, which permits a swift comparison of t-SNE plots constructed with diverse gene and/or miRNA lists, thereby enabling the visual identification of varying genetic patterns based on the chosen lists. Additionally, the tool is highly effective for selecting the samples to be examined, which helps to effectively categorize the data for differential genetic analysis.

### 2.3 Statistical analysis

Along with the morphing projections technique, the tool features statistical functionality, including the use of logistic regression to find genes or miRNA that best explain the differentiation between two groups selected by the user: miRNAs with higher estimated coefficients, as determined by the logistic regression model, have a greater impact on differentiation. This idea was originally introduced by Clark et al. in 2014 (13), in which they aimed to obtain the “characteristic direction” vector in the multidimensional space, and then to select the main components. However, given the limited number of samples in this case, it is crucial to take additional steps to verify the results. To this end, ANOVA tests are conducted to evaluate the statistical significance of the difference in expression level of the miRNA in the two groups.

## 3 Results

**Figure 1:**
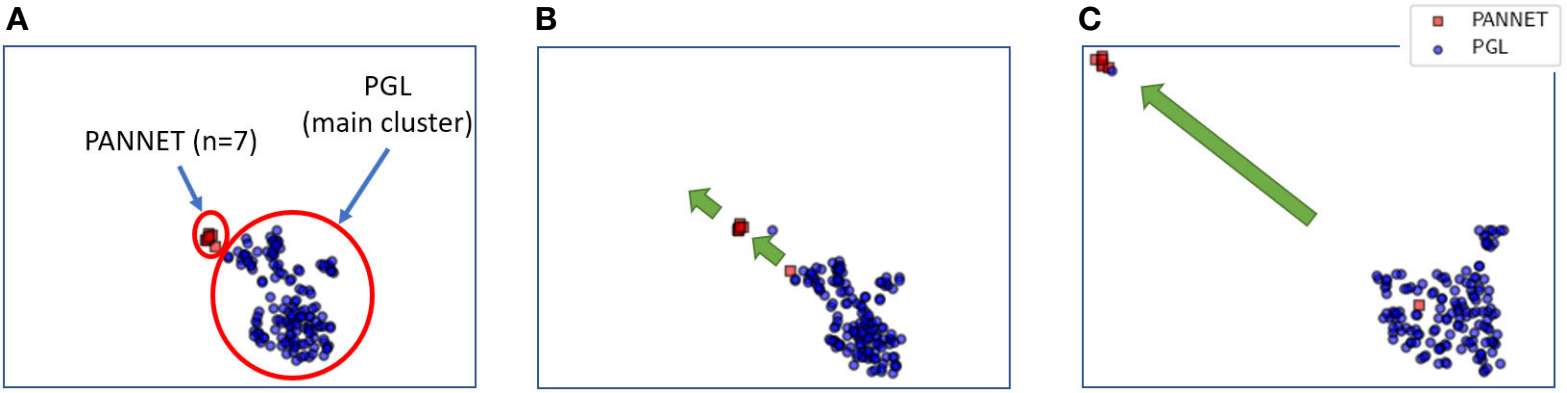
Smooth transition of t-SNE maps from gene expression profile **(A)** to miRNA expression profile **(B, C)**. In the gene expression profile **(A)**, PANNET samples cluster with PGL samples. However, as the weight of the t-SNE map shifts from gene expression to miRNA expression **(B, C)**, the PANNET samples move away from the PGL samples.

The morphing projections tool was used to analyze the PANNET and PGL included in TCGA. Smooth animated transitions from gene expression and miRNA expression *t*-SNE maps revealed a remarkably different genetic behavior of 7 samples that had been originally labelled as pancreatic adenocarcinoma. In the gene expression *t*-SNE view, these samples were grouped with PGL; however, when weight was given to a miRNA expression t-SNE view, they were found to move away. This suggests a similar gene expression profile, but different miRNA profile, as shown in **Figure 1**.

**Figure 2:**
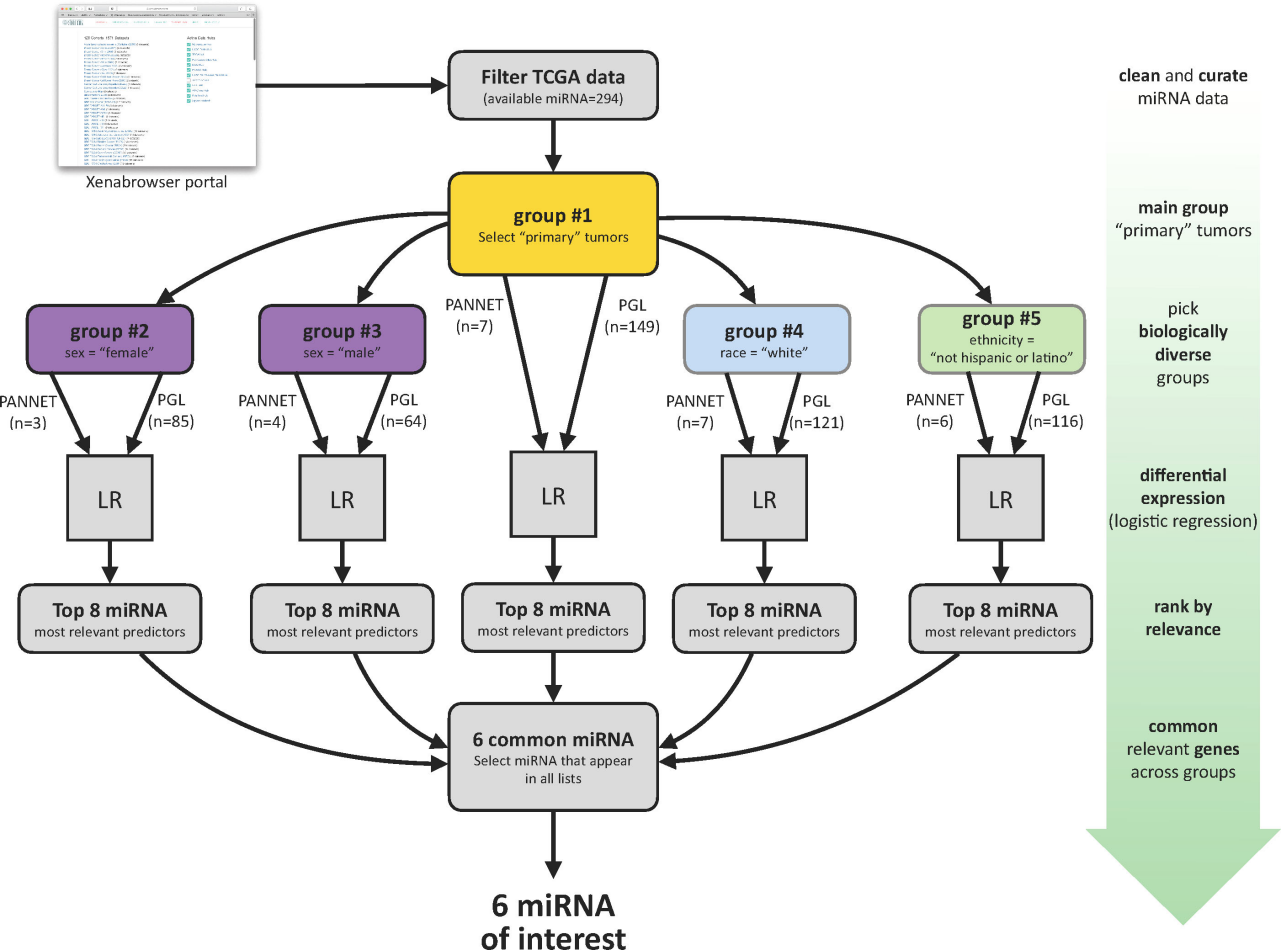
Summary of the data segregation and analysis pipeline for analysis between PANNET and PGL.

To further investigate this differential expression, data was divided into five groups as shown in **Figure 2**. First, samples not classified as primary tumors (those with TCGA codes not ending in -01) were eliminated to form the main analysis group #1. Groups #2 and #3 were separated based on gender, with “male” and “female” samples analyzed separately to prevent bias due to unequal proportions of both genders in the PGL set (64 vs. 85). Similarly, groups #4 and #5 were created using race “white” (all 7 available PANNET samples are “white”) and ethnicity “not hispanic or latino” (6 of the 7 PANNET samples belong to this ethnicity, except one that is unclassified).

**Figure 3:**
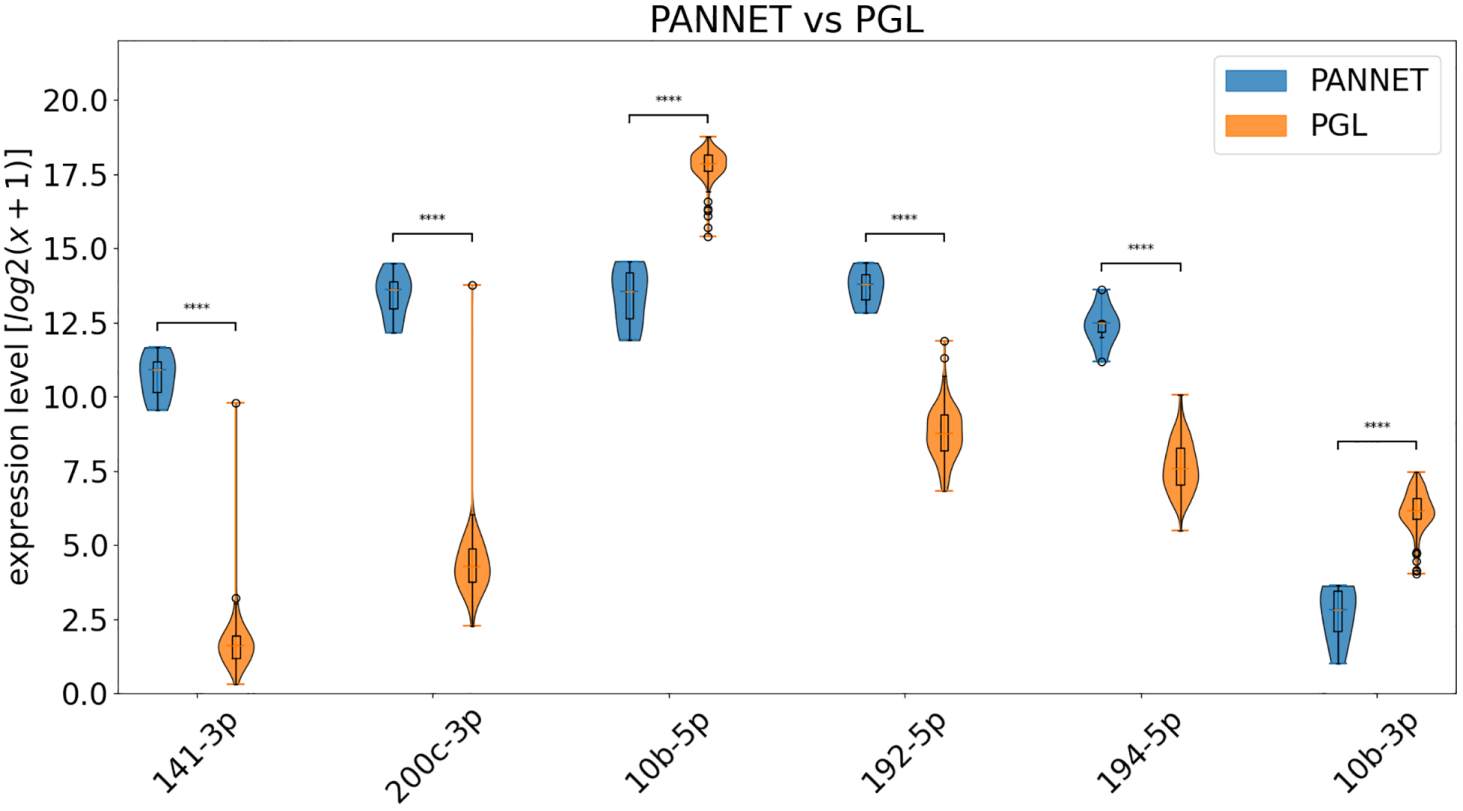
Violin diagrams showing the differential expression of the indicated miRNAs in PANNET and PGL included in the TCGA database. **** = p ≤ 0.0001.

A logistic regression was conducted in each group to identify the most significant miRNAs, as explained in section 2.3, and ANOVA tests were performed to determine the statistical significance. As a result, six miRNAs (-141-3p, -200c-3p, -192-5p, -194-5p, -10b-5p and -10b-3p) were repeatedly found among the top eight positions in all lists and were selected for further analysis (**Table 1** and **Figure 3**). Although more miRNAs could be considered, the six selected adequately explain the difference between both groups.

**Table 1:** Selected miRNAs of interest.

**miRNA**	**PANNET** **(mean ± std)**	**PGL** **(mean ± std)**	**ANOVA** **(p-value)**
hsa-miR-141-3p	10.7 ± 0.76	1.66 ± 0.87	p < 0.0001
hsa-miR-200c-3p	13.4 ± 0.80	4.33 ± 1.09	p < 0.0001
hsa-miR-10b-5p	13.4 ± 1.0	17.8 ± 0.50	p < 0.0001
hsa-miR-192-5p	13.7 ± 0.6	8.78 ± 0.87	p < 0.0001
hsa-miR-194-5p	12.4 ± 0.72	7.65 ± 0.86	p < 0.0001
hsa-miR-10b-3p	2.65 ± 0.96	6.17 ± 0.65	p < 0.0001

Expression levels are given as log2(RPM+1).

To validate these data, we used the following datasets: GSE29742 (14), which includes miRNA data from fresh-frozen samples of 48 PGL: 37 pheochromocytomas and 11 paragangliomas; and GSE73367 (15) consisting of miRNA data from fresh-frozen samples of PANNET (n=50). By incorporating these datasets into our analysis, we also aimed to address the limitation of having only 7 PANNET samples. However, the use of various methodologies and normalization protocols complicates direct comparisons between different datasets (16). To address this issue, we applied percentile normalization to the data. In the absence of control samples, each value’s percentile was determined based on the full expression levels across all samples in the dataset. This approach allowed us to identify miRNAs that are overexpressed (high percentile values) or underexpressed (low percentile values) relative to the rest of the data.

**Figure 4:**
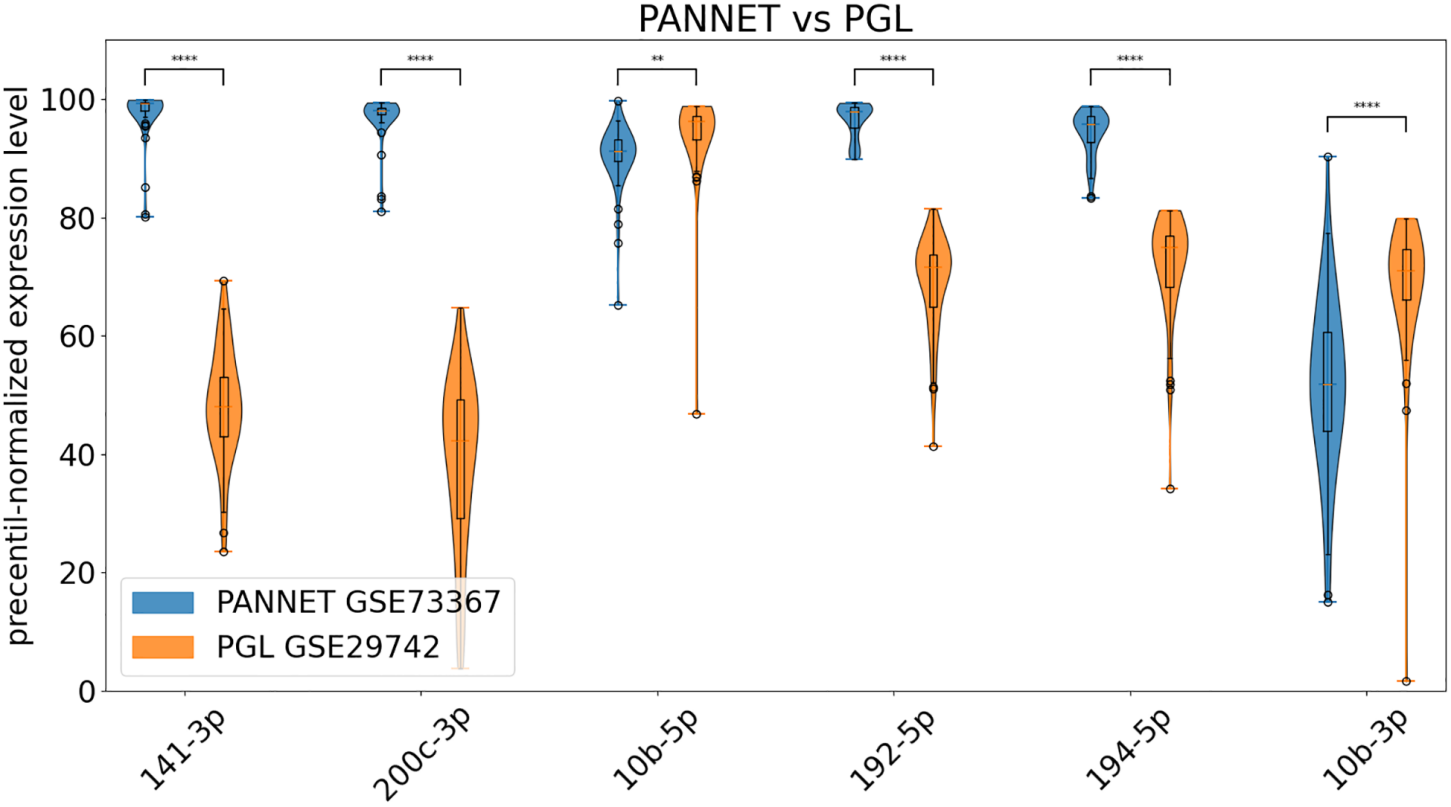
Percentile-normalized expression levels of the indicated miRNAs in PANNET, PGL from datasets GSE73367 and GSE29742 (** = p ≤ 0.01, **** = p ≤ 0.0001).

Results are shown in **Figure 4** and clearly confirm our findings for miR-141-3p, -200c-3p, -192-5p and -194-5p, which are clearly overexpressed in PANNETs versus PGL. Additionally, miRNA-10b-5p and miR-10b-3p are overexpressed in PGL compared to PANNET, and although the magnitude of the difference is lower, it is still statistically significant.

## 4 Discussion

Our research has uncovered a remarkable similarity at the genetic level between PANNETs and PGLs, yet a striking dissimilarity in their miRNA expression profiles. We have identified 6 key miRNAs that can effectively distinguish between the two types of tumors, addressing a crucial need for more accurate diagnosis in the medical community.

We report here that 4 of the 6 identified miRNAs are elevated in PANNETs compared to PGLs. Interestingly, these microRNAs are members of two miRNA families: miR-200c/141-3p and miR-192/194. miRNA families often share similar sequences and structures, indicating a shared function (17). The miR-200c/141-3p family, located on chromosome 11p, has a complex role in cancer, acting as either an onco-miRNA or tumor suppressor depending on the type of cancer. It regulates epithelial-mesenchymal transition through targeting ZEB1/2 (18, 19) and has been linked to pro-apoptotic and anti-proliferative effects in several cancers, yet it also promotes tumor growth, invasion, and migration in others (20). High expression of miR-200c is associated with poor overall survival in gastric and non-small cell lung carcinomas (21, 22), but with better prognosis in ovarian and bladder cancer (23, 24). In pancreatic neuroendocrine cells, the promotion of beta cell survival by miR-200c has been demonstrated to be essential (25, 26). Meanwhile, miR-141 has demonstrated tumor suppressive effects in multiple cancers, including pancreatic adenocarcinomas (27). The role of miR-192/194 family in cancer is complex and still debated, with evidence supporting both oncogenic and tumor-suppressive effects (28). miR-192 has been shown to inhibit tumor angiogenesis (29), while miR-194 suppresses proliferation, migration, and metastasis and induces apoptosis in cancer cells (30–33). The functional significance and mechanisms of action of these two families of miRNAs in neuroendocrine tumors, such as PANNET and PGL, remains unclear. Further research is needed to fully understand their impact on these tumors.

Our study also showed that PGLs express higher levels of both the -3p and -5p strands of the miR-10b duplex compared to PANNETs. Traditionally, only one strand of the miRNA duplex, known as the functional strand, was thought to target specific mRNAs, while the other strand was degraded. However, recent research has revealed that both strands of the duplex can be functional (34), which could account for the accurate detection of both miR-10b strands in both PANNET and PGL. One study has reported that miR-10b can differentiate PGL from neuroblastoma (35). Our results further support its potential as a biomarker for differentiating PANNET from PGL. miR-10b has been shown to be de-regulated in several types of cancer, but it is still unclear if it is causally related to cancer initiation or progression (36–39). To date, many studies have shown that miR-10b could be involved on metastasis in a wide range of cancer types supporting a role for this miRNA in cancer progression (36).

Retroperitoneal PGLs, although rare, can occur in the vicinity of or within the pancreas, resembling primary pancreatic lesions. To date, literature records have documented approximately 56 peripancreatic PGLs (2, 6, 40–46). Distinguishing between PANNETs and PGLs is crucial for appropriate patient management, both preoperatively and postoperatively. An accurate diagnosis of PGLs is essential for preoperative planning as previous research has shown that perioperative mortality and morbidity can be high if a PGL is diagnosed during surgery. This is because induction of anesthesia and manipulation of the tumor during surgery can trigger catecholamine release. In addition, the majority of PGLs are benign; however, the risk of developing metastatic disease is higher in abdominal PGLs, with rates ranging from 11-36%. Currently, there are no reliable markers to predict malignancy, except for the presence of metastases. This highlights the need for follow-up for patients with PGLs as metastatic disease can appear years after diagnosis. Singhi et al (6), reported that 3 of 9 (33%) of patients diagnosed with peripancreatic PGL developed metastases within 5 years of surgery, and 2 of them died because of their disease. In another report (47), 13.9% exhibited a recurrence or widespread disease and one patient died 48 months following diagnosis. Additionally, it is important to note that more than 40% of abdominal PGLs carry a germline pathogenic gene variant, which has significant clinical implications for personalized surveillance and treatment and for the patient’s family.

Peripancreatic PGLs not only confound diagnosis through their clinical presentation and imaging characteristics, but also pose a significant challenge for even the most experienced pathologists. On surgical resection, both neoplasms are usually solitary, well-demarcated lesions with a fibrous border. In contrast to the peripancreatic PGLs, PANNETs are typically located inside the pancreatic parenchyma. However, parenchymal invasion was observed in many cases of peripancreatic PGLs with loss of zellballen architecture in these areas making accurate assessment of tumor location challenging. Moreover, PANNETs may display a variety of architectural patterns, including nested and solid growth, which may resemble a zellballen architecture.

By immunohistochemistry, both PGLs and some well-differentiated PANNETs demonstrate S-100-positive sustentacular cells. PANNETs are generally positive for cytokeratins AE1/AE3 and CAM 5.2, whereas PGLs are distinctly negative. Nevertheless, a more comprehensive panel of immunohistochemical stains, including cytokeratins, Vimentin, GATA-3 and/or Pax 8, has been recommended for accurate differentiation between the two types of tumors (6, 48, 49) as a single defining feature is not sufficient. In the absence of clear clinical indications, a diagnosis of a primary pancreatic neoplasm may be more likely than PGL. Ultimately, it is the responsibility of the pathologist to arrive at a definitive diagnosis of PGL. Our findings of using miR-200c/141-3p, miR-192/194 and miR-10b levels as potential biomarkers offer a promising solution to this challenge and hold significant implications for accurate diagnosis of PANNET and PGL.

### 4.1 Limitations of this study

The TCGA data, while balanced in terms of gender representation, lacks diversity in terms of race and ethnicity, as most samples are from the ‘white’ or ‘non-hispanic-or-latino’ groups. This could introduce bias, especially in the case of PANNET where all samples come from these groups. Moreover, the TCGA study only included data from a small number of PANNET samples (7), further limiting its representativeness. By incorporating additional datasets into our analysis, we aimed to address the constraint of a small sample size. However, it’s important to acknowledge that these validation datasets lack clinical data, preventing us from exploring variations in the expression levels of the identified microRNAs across different stages of the disease or determining whether the tumors with different behaviours (benign or malignant) exhibit distinct miRNA expression profiles. Therefore, it is crucial to validate the findings of this study using larger sample sizes that encompass diverse racial and ethnic backgrounds and include comprehensive clinical data to facilitate a broader analysis.

## 5 Conclusions

This study uncovered six key microRNAs (miR-10b-3p, miR-10b-5p, and the miRNA families miR-200c/141 and miR-194/192) that can effectively differentiate peripancreatic PGLs from PANNETs. These findings suggest that measuring the levels of these miRNAs in tumor tissue samples could serve as potential biomarkers for more accurate diagnosis, potentially improving the standard of care for patients.

## Data availability statement

Publicly available datasets were analyzed in this study. This data can be found here: https://portal.gdc.cancer.gov and in https://www.ncbi.nlm.nih.gov/ for the Gene Expression Omnibus: GSE29742 and GSE73367.

## Author contributions

JE and ID designed and managed the data study. JE, ID and DG performed the data curation and analysis. The biomedical study was designed and managed by M-DC and NV. TC performed bioinformatics analysis. JE, ID, M-DC and NV wrote and reviewed the paper. All authors contributed to the article and approved the submitted version.
